# Predicting pattern formation in embryonic stem cells using a minimalist, agent-based probabilistic model

**DOI:** 10.1038/s41598-020-73228-4

**Published:** 2020-10-01

**Authors:** Minhong Wang, Athanasios Tsanas, Guillaume Blin, Dave Robertson

**Affiliations:** 1 Usher Institute, Edinburgh Medical School, The University of Edinburgh, Edinburgh, EH16 4UX UK; 2 Centre for Regenerative Medicine, Institute for Regeneration and Repair, The University of Edinburgh, Edinburgh, EH16 4UU UK; 3 School of Informatics, The University of Edinburgh, Edinburgh, EH8 9AB UK

**Keywords:** Cell biology, Computational biology and bioinformatics, Stem cells

## Abstract

The mechanisms of pattern formation during embryonic development remain poorly understood. Embryonic stem cells in culture self-organise to form spatial patterns of gene expression upon geometrical confinement indicating that patterning is an emergent phenomenon that results from the many interactions between the cells. Here, we applied an agent-based modelling approach in order to identify plausible biological rules acting at the meso-scale within stem cell collectives that may explain spontaneous patterning. We tested different models involving differential motile behaviours with or without biases due to neighbour interactions. We introduced a new metric, termed stem cell aggregate pattern distance (SCAPD) to probabilistically assess the fitness of our models with empirical data. The best of our models improves fitness by 70% and 77% over the random models for a discoidal or an ellipsoidal stem cell confinement respectively. Collectively, our findings show that a parsimonious mechanism that involves differential motility is sufficient to explain the spontaneous patterning of the cells upon confinement. Our work also defines a region of the parameter space that is compatible with patterning. We hope that our approach will be applicable to many biological systems and will contribute towards facilitating progress by reducing the need for extensive and costly experiments.

## Introduction

Developmental patterning is the process whereby a population of cells initially seemingly homogenous differentiates in a spatio-temporally coordinated manner to generate segregated domains of distinct cell fates. During mammalian development, early patterning events break symmetry and define the future body axes of the embryo^1,2^.

Decades of research in model animals have permitted to uncover molecular players responsible for the patterning of the mammalian embryo. These include diffusible signals secreted by the cells such as Nodal, BMP4, Wnt and their respective inhibitors engaged in a complex molecular interplay that involves many feedback mechanisms^3,4^. Yet much remains to be understood about how the precise positioning of individual cell fates are coordinated with morphogenetic events in space and time.

In recent years, new insights have been gained with embryonic stem cells (ESCs) in vitro. ESCs are a population of cells endowed with the capacity to self-renew and differentiate in vitro into all somatic cell types as well as into the germ cell lineage. When ESCs are geometrically confined on adhesive areas of specific sizes and shapes (micropatterns), ESCs self-organise to form patterns of cell fates reminiscent of the organisation of cell fates found in vivo^5–8^. One advantage of in vitro systems is that they bring the system to a level of complexity that makes it amenable to interpretation and computational modelling. For example, ESCs-based in vitro systems have made it possible to investigate signalling dynamics using both experimental perturbations and computational modelling approaches to better understand how distinct domains of cell fates are specified over time^9–11^.

One aspect that remains relatively unexplored is how distinct group geometries and local cellular movements may affect the boundaries between domains of cell fates initially specified by signalling molecules. We recently proposed that mesoscale rearrangements may be important based on our own observations made with mouse ESCs (mESCs)^4^.

Indeed, we recently showed that when a heterogeneous population of ESCs are confined on small (30 000 μm^2^) elliptical shapes, a subpopulation of cells marked by brachyury (T) becomes restricted to the tips of the ellipse. This is interesting because (1) the location of brachyury marks the proximo-posterior side of the embryo in vivo and announces the onset of gastrulation. The precise positioning of T+ cells is therefore of paramount importance during development. (2) T+ cells are found in a region where epiblast curvature is particularly pronounced raising the possibility of a role for this geometric feature in the process.

It is known that the domain of expression of brachyury is initiated by diffusible signals such as Wnt and Nodal. Now at the cellular level, heterogeneity in expression is observed within the T+ domain. Evidence in the chick suggest that local community effects and mechanical rearrangement operate a selection of the cells that eventually ingress into the streak^12^. Therefore, the properties acquired by the cells as they become T+ combined with the local geometry of the embryo may contribute in the precise patterning of T+ cells.

Here we decide to employ a computational approach in order to quantitatively explore various set of logical rules that could explain the patterning of T+ cells in response to geometrical confinement. Computational models coupled with experimental data provide approaches to analyse, predict, and understand cell behaviours^13^. Hence, developing a modelling strategy to investigate pattern formation at a high level has the potential to provide a practical approach towards delivering new physiological insights.

A wide variety of modelling approaches exist to tackle multi-cellular systems. For example, in recent years, multi-scale modelling approaches have been successfully employed to recapitulate in-silico the cell fate decisions and morphogenetic events occurring during chick gastrulation^14,15^ or during the pre-implantation stages of mouse development^16,17^.

Here we employ an agent-based modelling approach to investigate the rules at the micro-level that lead to ordered pattern at the macro-level. Agent-based models are often state-based and defined in terms of logic-based rules, and have been particularly successful in settings where the primary scientific question is about collective activities through discrete cell states or fates^18^. d’Inverno and Saunders illustrated that compared to cellular automata approach, in which the grids stand for cells, agent-based system is the more appropriate approach to model the behaviour of ESCs to understand the process of self-organisation because of its higher degree of freedom with modelling the cells as agents that can move on the top of the environment^19^. In agent-based models, each cell contains a unique identifier and internal counter which allows the tracking of the cells. Moreover, with agent-based models, the information exchange between cells and their neighbouring cells, cells and their environment can be modelled more naturally.

Thus, using agent-based modelling, we generated and tested a repertoire of logical rules for their ability to recapitulate pattern formation upon geometrical confinement. We found that differential motility biased by cell density is sufficient to explain how T+ cells become restricted to the tips of elliptical shapes. Using a new metric for the quantification of patterning, we identified the region of the parameter space that is compatible with proper patterning in this context. Altogether, our work provides a generic, parsimonious and testable mechanism for the emergent patterning of cells that are initially randomly distributed as well as a framework for developing and testing models of pattern formation in geometrically confined groups of cells.

## Materials and methods

### Empirical data

**Figure 1:**
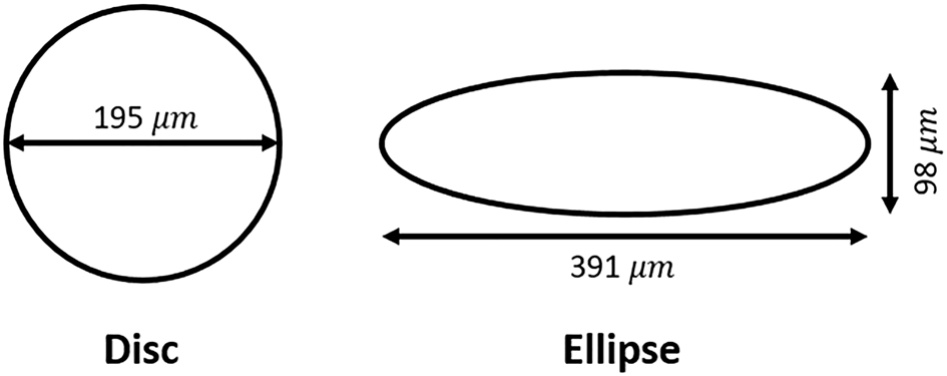
The dimensions of disc and ellipse micropatterns.

We used the data previously described in Blin et al.^4^ mESCs were cultured in LIF/Serum conditions. These conditions are known to maintain the cells in a dynamic equilibrium comprised of heterogeneous cell states including both undifferentiated cells and cells primed for differentiation^20^. Micropatterning was applied to control the precise spatial deposition of cell adhesive molecules onto the surface of the culture dish. This technique makes it possible to control the biochemical environment of the cells while at the same time providing precise geometrical confinement. A detailed video protocol of the method employed in Blin et al. is available at https://www.jove.com/t/59634/mapping-emergent-spatial-organization-mammalian-cells-using^21^. Briefly, we have 186 images for disc micropatterns and 152 images for ellipse micropatterns. Each images contains one cell colony that was captured after 48 hours from the initial seeding of the stem cells. The dimensions of disc and ellipse are shown in Fig. 1. The radius of the disc is 97.5 μm; the semi-major axis of the ellipse is 195.5 μm and the semi-minor axis of the ellipse is 49 μm.

In this study, we focus on studying the pattern formation observed in T+ and T− cells on disc and ellipse micropatterns. T+ cells are early differentiated cells as they are marked by Brachyury (T); T− cells are naïve cells, which means they have not started differentiation yet. On a cellular level, specific pattern formation of T+ and T− cells can be observed 48 hours after seeding cells randomly on the disc and ellipse shaped micropatterns. On average 6 cells were seeded on the disc or ellipse micropatterns, which means theoretically cells can only survive within these restricted areas. However, it may be possible that a few cells might migrate out of these restricted areas in practice. This might be due to the degrading of the hydrophobic substrate, proteins attaching to the hydrophobic regions, or matrix secreted by cells etc. We do not include the cells migrated out of the micropatterns in our modelling since we assume it is a result of uncontrollable noise in the wet lab. In addition, we selected cells within the micropatterns that are more than 2 μm inside the border to obtain a clear cut at the border of the micropatterns. For disc micropatterns, we selected cells within 95.5 μm from the centre of the disc; for ellipse micropatterns, we selected cells within the ellipse with the semi-major axis as 193.5 μm and semi-minor axis as 47 μm.

On an aggregated level having multiple cell colonies, after 48 hours from random seeding for disc micropatterns, T− cells preferentially stay at the centre of the disc while T+ cells preferentially localise at the edge of the disc. For ellipse micropatterns, T+ cells preferentially localise at the tips of the ellipse only, while T− cells preferentially stay at the centre of the ellipse.

**Figure 2:**
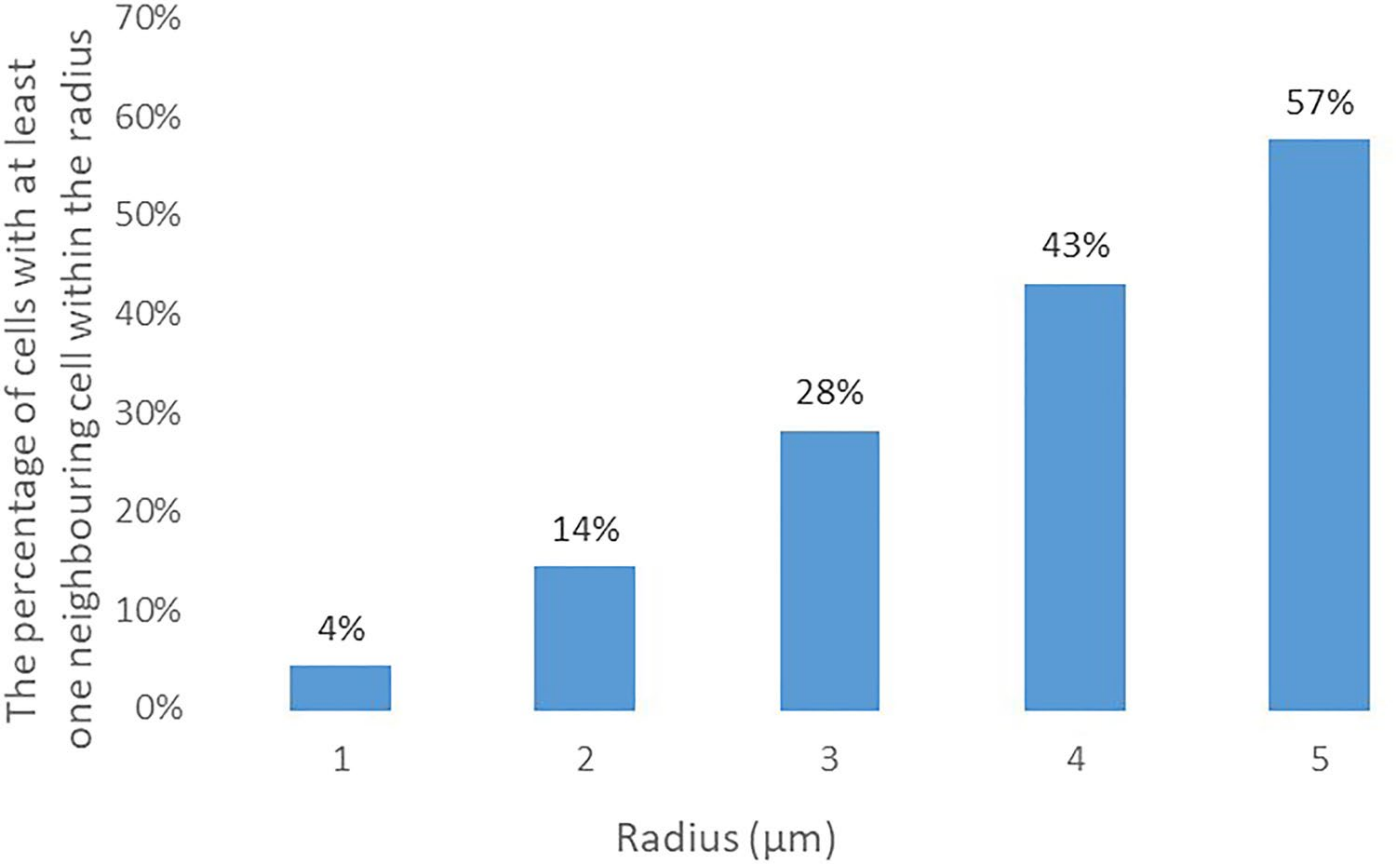
The percentage of cells with at least one neighbouring cell and the radius (μm).

As we only consider the 2D pattern we observed, we extracted the cells’ locations (described by x and y coordinates) based on the centres of the nuclei. Based on the 2D locations, we counted the number of neighbouring cells for each cell within a certain radius. We calculated the percentage of cells with at least one neighbouring cell within a certain radius (as shown in Fig. 2). For example, 14% of cells have at least one neighbouring cell within 2 μm.

### Model construction

**Figure 3:**
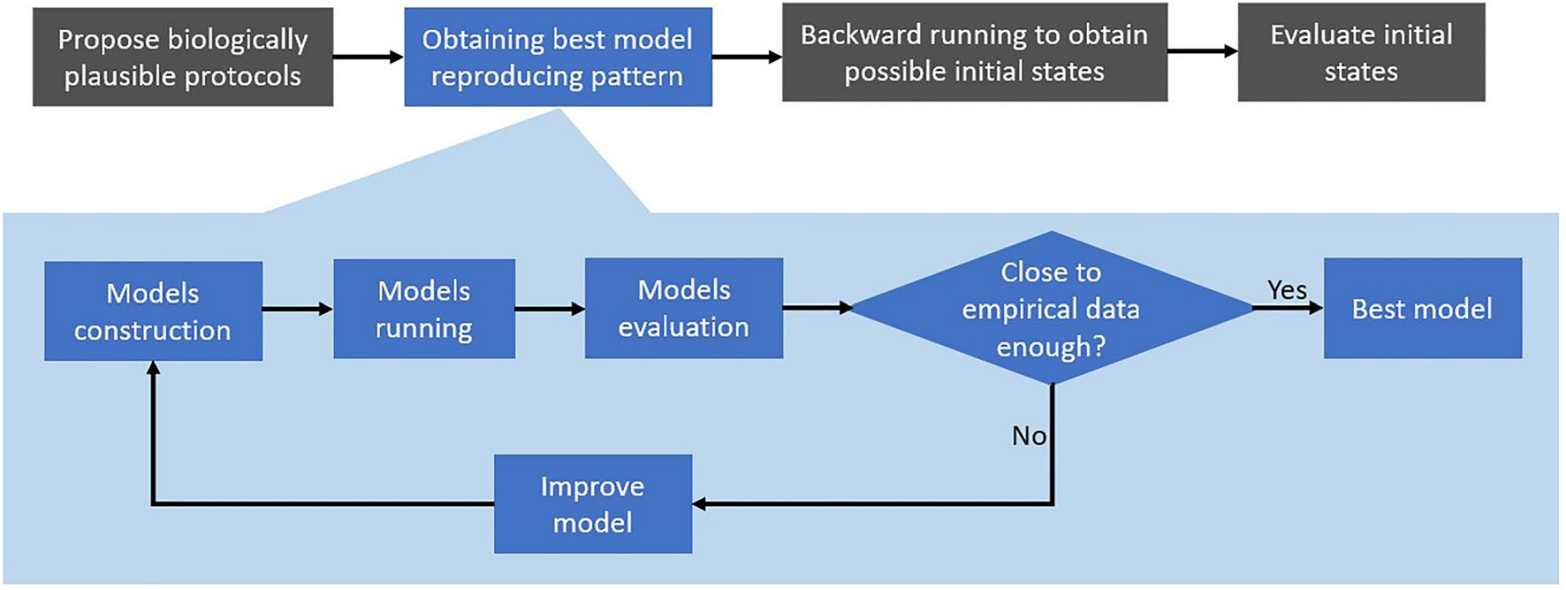
Workflow of this study.

Figure 3 presents the workflow in this study. We first propose biologically plausible rules of cell motility which may lead to the resulting pattern formation. We next constructed a set of models to test all combinations of these possible rules (see below for details) as evaluated the models through a novel metric that measures the distance between models and empirical data. With inversing models, we can obtain possible initial states providing information to biologist about seeding cells.

We applied agent-based models for self-organised pattern formation in ESCs from a population level. We have previously described the model structure^22^. Models were constructed in NetLogo 6.0.4, an open sourced environment specifically designed for agent-based modelling. The code is available online at https://github.com/MinhongW/ESCs_models. In short, there are two types of agents standing for cells and environments with their own parameters (parameters are listed in Table 1). The models are lattice-based as environment agents are a set of square patches that cell agents can move on top of, one patch stands for 1 μm. The models are state-based with 192 timestamps representing 48 hours in real life, i.e. we have measurements every 15 minutes which correspond to the timing resolution used (timestamps).

**Table 1:** The list of terms and corresponding descriptions of different types of agents.

Agent types	Terms	Descriptions
Environment	Inside/outside	Inside or outside of the micropattern
	Occupied/Not occupied	Stands for the patch is occupied by a cell or not
Cell	Location	The location of the cells’ centre point (consist of *x* and *y*)
	Closest distance to neighbouring cells	The closest distance cells can be, same value for T+ and T− cells (set as 2 μm due to projecting 3D in real world to 2D in models)
	Cell type	T+ or T− cells
	Mean velocity (*v*)	The mean velocity of cells (As the value of velocity have been measured experimentally, we set 100 μm/h for T+ cells and 40 μm/h for T− cells^23,24^)
	Velocity ratio	The ratio of mean velocity to obtain the actual velocity, more details in Eq. (2)
	Directional persistence time	The time that migratory cells spend without changing direction (105 min for T+ cells and 15 minutes for T− cells)^25^
	Direction	The heading direction of cells
	Sensing radius (*R*)	How far away the neighbouring cells they can sense for mechanical forces
	Standard deviation (*σ*)	For generating the noise of receiving mechanical forces in real life (e.g. from different shape of the cells or noise caused by some diffusible signals we do not understand yet)
	Angle change (*α*)	The angle cells change when they reach the border of the micropattern (we tested 10, 20, 30, and 40 degrees in this study)

The aim of the study is to construct a minimal model to investigate the contribution from cell motility to pattern formation, therefore, we omit the contribution from different shapes of cells, cell differentiation, division and apoptosis. Similarly, we do not include additional physical rules between the cells and their environment to simplify the resulting model.

The biologically plausible rules we proposed are:

1. Different velocity of T+/T− cells. Based on former experimental studies from Turner et al.,^23^ and Phadnis et al.,^24^, T + cells have higher speed than T− cells. The velocity values we set in our models are based on the mean velocity from previous studies.
2. T + cells have higher directional persistence time (inspired by the study of Mori et al.,^25^).
3. Directional movements decided by neighbouring cells (based on the empirical observations from wet lab that T+ cells move away from neighbouring cells and T− cells move toward to neighbouring cells).We hypothesize that cells immigrating direction depends on the forces they received from their neighbouring cells within a distance (sensing radius *R*). We calculate the force according to
  1 $$\begin{array}{*{20}c} {F_{x,y} \sim \frac{1}{{D^{2} }}} \\ \end{array}$$
  where *F*_*x,y*_ is the force with *x* and *y* representing the direction of the force in the two dimensional hyper-plane. *D* is the Euclidean distance between cells. T+ cells receive pushing forces from their neighbouring cells, while T− cells receive pulling forces from their neighbouring cells. Then we sum up the forces to get the direction of cells. The final immigration direction is generated by a normal distribution of the final sum force with a specific standard deviation (*σ*). Normal distribution was used as noise generator.
4. Border effect of cells (based on the assumption that cells tend to stay within the micropatterns instead of escaping from these constraint areas). When a cell reach the border of the constraint area, which means with current immigrating direction and speed the cell is tempting to immigrate to somewhere outside of the constraint area in next state, this cell will change its immigrating direction by a small angle (*α*). By comparing the current direction and the direction to the closest patch outside of the constraint area, the cell will decide the angle change is clockwise or counter clockwise.

**Figure 4:**
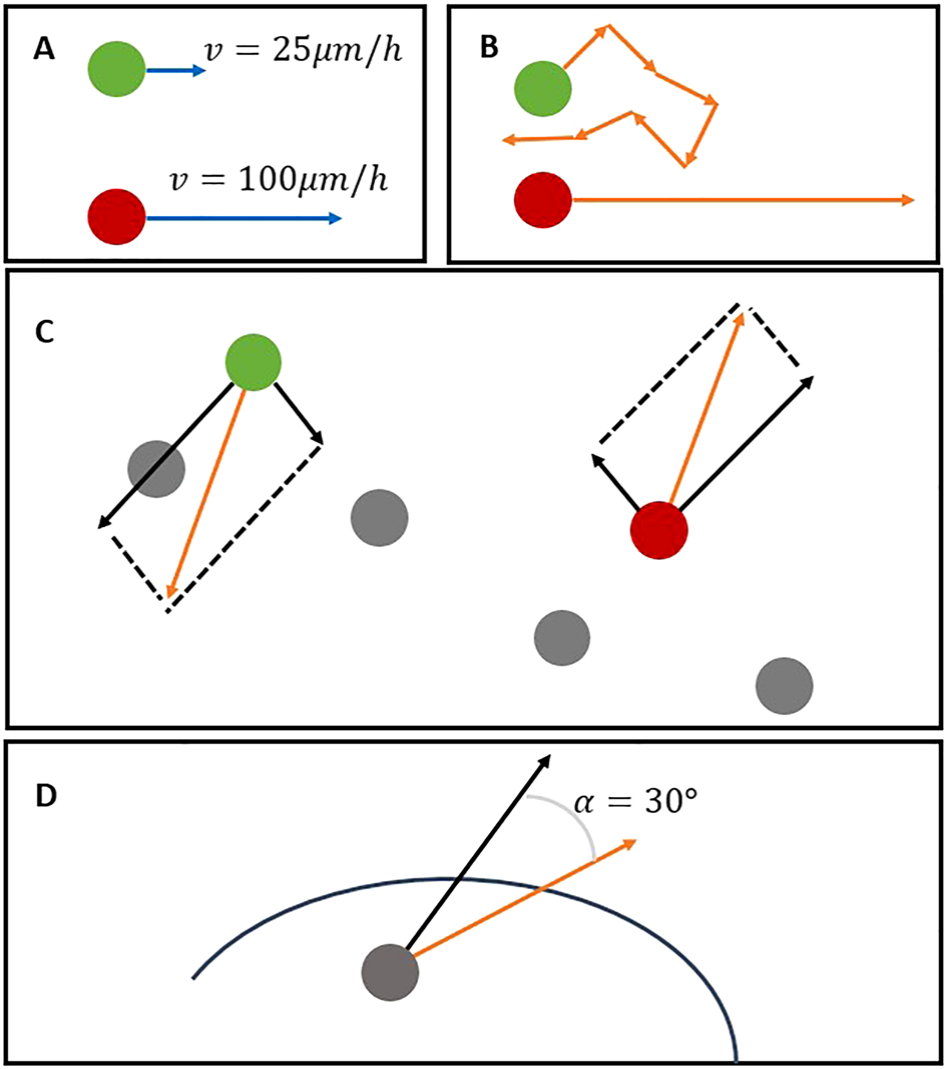
Illustration of protocols with green circles stand for T− cells, red circles stand for T+ cells, and grey circles stand for both T+/T− cells. (**A**) different velocity of T+/T− cells, blue arrows stand for velocity (without direction); (**B**) T+ have higher directional persistence time, orange arrows stand for immigrating direction; (**C**) directional movements decided by neighbouring cells for T+/T− cells, black arrows stand for the forces from neighbouring cells calculated according to Eq. (1), orange arrows stand for the immigrating direction which decided by the sum of the forces; (**D**) border effect of cells that will change a small angle when they reach the border of the constraint areas. Black arrows stand for the old direction, while orange arrows stand for the final direction.

Figure 4 shows the diagram illustrating these rules. In this study, we tested all combinations of these four rules with 16 agent-based models. The list of the models numbers with corresponding rules is shown in Tables 2 and 3.

**Table 2:** Four proposed biologically plausible rules.

Rules	Descriptions
i	Different velocity
ii	Different persistence time
iii	Directional movements
iv	Border effect

**Table 3:** Models and their corresponding rule combinations.

Model	Rules
1	None
2	i
3	ii
4	iii
5	iv
6	i + ii
7	i + iii
8	i + iv
9	ii + iii
10	ii + iv
11	iii + iv
12	i + ii + iii
13	i + ii + iv
14	i + iii + iv
15	ii + iii + iv
16	i + ii + iii + iv

Subsequently, based on the previous rule that cells get the direction from neighbouring cells and move with fixed speed, we improved the selected best models by adjusting cell velocity by neighbouring cells, hence, cells do not always migrate with the same velocity. In addition to the previous rule of directional movements, we calculate the ratio of actual velocity and previous mean velocity based on the sum of forces divided by the sum of the magnitude of the forces. *v* is the previous mean velocity of this type of cell. *v*_*actual*_ is the actual speed of cell migration.

2 $$\begin{array}{*{20}c} {v_{actual} = v \times \frac{{\sum F_{x,y} }}{{\sum \left| {F_{x,y} } \right|}}} \\ \end{array}$$

### Parameter optimisation through grid search

Based on the model with improved rules, we tested a set of parameters. Because the number of free parameters is not big, we applied grid search for parameter optimisation to test all combinations of the values of these free parameters. The free parameters we tested are different sensing radius (*R*) for getting neighbouring cells and standard deviation (*σ*) for generating the final direction (as listed in Table 4). According to the results illustrated in former experiments of growing T+/T− cells in confined areas^4^, T+ and T− cells shows different preferential localisation by taking 50 μm and above as radius for neighbourhood on disc and ellipse micropatterns. Considering the size of the micropatterns, we tested 25, 50, 75, and 100 μm for sensing radius in our models.

**Table 4:** The list of possible values of the parameters used in a grid search setting.

Parameters	Values for disc	Values for ellipse
Sensing radius (*R*)	25, 50, 75, 100	25, 50, 75, 100
Standard deviation (*σ*)	1, 3, 5	1, 3, 5

Following parameter optimisation, we tested applying different sensing radius (*R*) and different standard deviation (*σ*) for T+ and T− cells separately. Hence, T+ and T− cells can have different optimised value for these parameters. Again, we applied grid search for parameter optimisation by testing all combinations of the potential values. We get the model with the best performance by applying the metric explained in the following section.

### Model validation and a new evaluation metric

**Figure 5:**
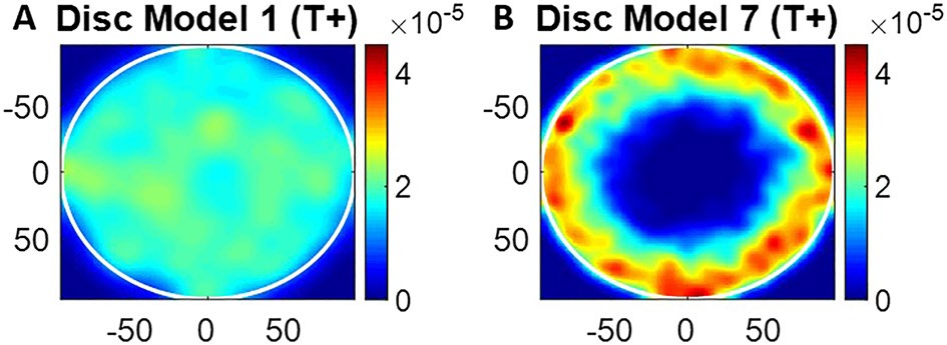
Density plot showing the results from (**A**) model 1 and (**B**) model 7 on disc micropatterns for running models 100 times.

We tested related existing approaches to evaluate pattern formation in disc and ellipse models against empirical data. For example, we considered the earth mover’s distance^26^ (EMD), Kullback–Leibler divergence^27^ (KL divergence) and continuous rank probability score (CPRS) metrics (for distribution density of each grid point)^28^. We take output from Model 1 (random model) and Model 7 on disc micropatterns as example. Figure 5 shows the density plot of T+ cells distribution after running models 100 times. The results from Model 7 is closer to our desired pattern as T+ cells prefer to localise at the border on the disc. Table 5 shows the results of EMD, KL divergence and CRPS. Hence, for all these established approaches, the resulting outcome did not follow the visual impression we had from observing the resulting patterns, which motivated the development of a new approach that was tailored specifically to this application.

**Table 5:** The evaluation results of model 1 and model 7 based on existing metrics.

	Model 1	Model 7
EMD	5.4559	13.306
KL divergence	0.0465	0.4695
CRPS	5.00e−6	5.77e−6

**Figure 6:**
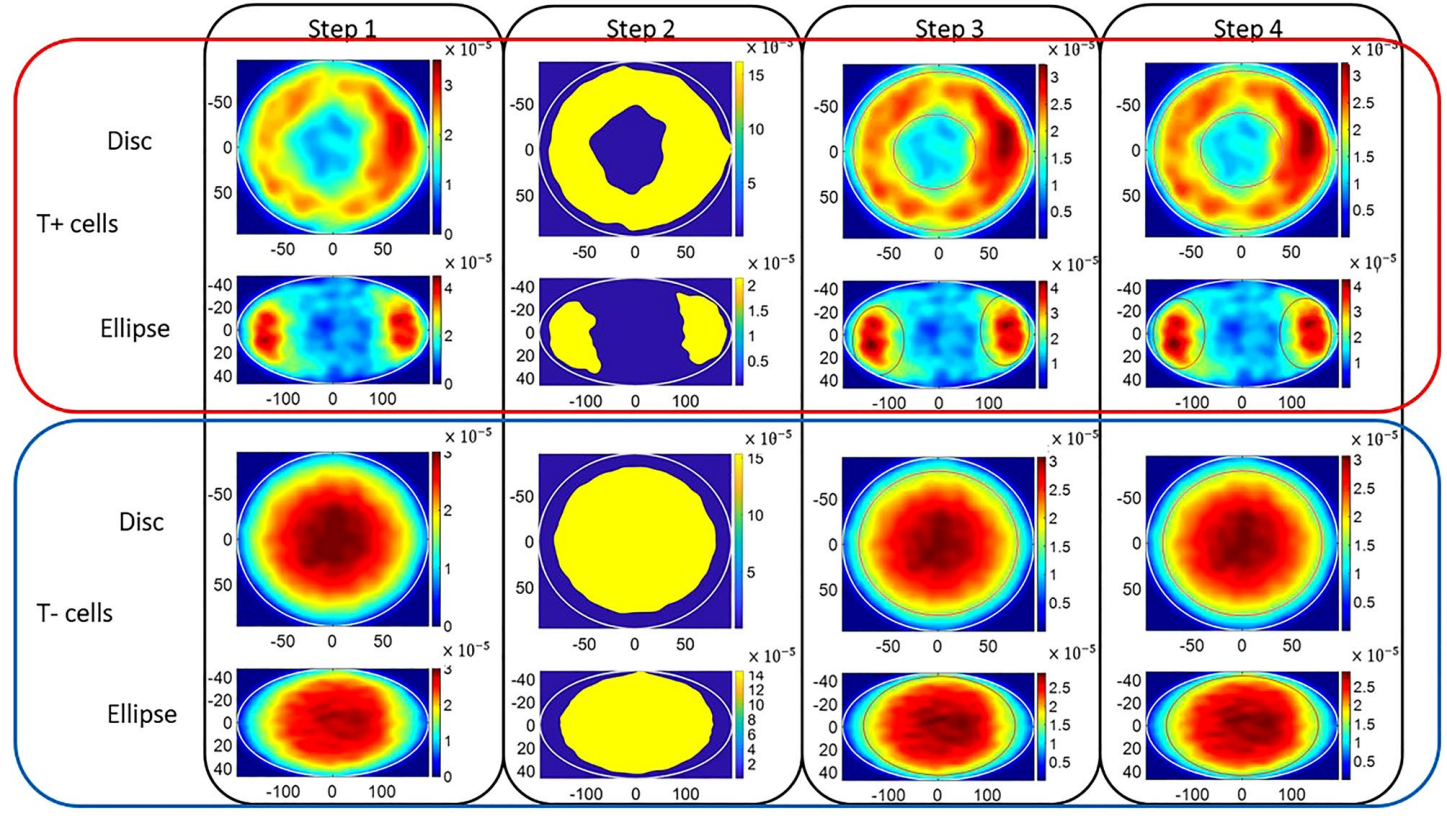
The process of getting the borders of evaluation of T+ cells and T− cells separately for disc and ellipse micropatterns from step 1 to step 4 accordingly. White lines stand for the border of the micropatterns. In step 1, the density maps of T+/T− cells on disc and ellipse micropatterns are provided. In step 2, the contour plots were generated by thresholding. In step 3, best fit circles/ellipses for the borders of HDA are shown in red lines. In step 4, the asymmetric problems are fixed.

We developed a new metric to quantify the difference in the constellation of the cell patterns between the model results and the empirical data, which we call the *stem cell aggregate pattern distance* (SCAPD). The metric calculates the distance between the total densities within high density areas (HDA) of aggregate cell cultures and takes T+ and T− cells into account separately. Figure 6 illustrates the process of getting the borders of high density areas for evaluation. The steps of getting the borders are:

- *Step 1* Getting density maps: We applied 2D kernel density estimation^29^ with Gaussian kernels to generate aggregate density maps of T+ and T− cell colonies separately. We applied Botev’s approach for density estimation and used their internal bandwidth estimate with default optionse^30^. We calculated the density over a 256 × 256 grid space, hence the resulting density estimate comprises 256 × 256 entries.
- *Step 2* Getting points by thresholding: Based on density maps, we collected a list of points marking the border of the areas by thresholding. The threshold T was calculated as the mean of maximum and minimum value of density of each grid point (*g*_*i*_), as shown below.
  3 $$\begin{array}{*{20}c} {T = \frac{{\max \left( {g_{i} } \right) + \min \left( {g_{i} } \right)}}{2}} \\ \end{array}$$
- *Step 3* Best fit circles/ellipses: Based on the points we marked, we generated the best fit circles or ellipses based on least-squares fitting^29^.
- *Step 4* Fixing asymmetric: Since the cells were seeded randomly and there is no fundamental reason to suggest the micropatterns should exhibit particular preference on either end (e.g. left vs right part of the ellipse), we do not believe cells would end up on a specific direction. Due to the symmetrical properties of disc and ellipse, we believe that the assessment of the disc micropatterns should be symmetric according to any arbitrary angle, and the pattern on ellipse micropattern should be symmetric according to x and y axis. For disc micropattern, we keep the radius of the best-fit circles and moved the centres to the centre of the disc micropattern (point(0,0)). For ellipse micropatterns, we use the maximum value for both semi-minor and semi-major axis of the best fit ellipses, and then we keep the absolute value of *x* of the centre of the best-fit ellipse as the mean of the absolute values of two *x* values, and *y* of the centre of the best-fit ellipses as 0.

After setting the borders, we calculated the total density within these areas for T+ and T− cells separately in empirical data on both disc and ellipse micropatterns. The total density is the sum of the density for the subset of the originally evaluated 256 × 256 grid points for the density which fall within the defined borders that we have set up for matching to ground truth. Hence, for empirical data, we got the total density within the area for both T+ and T− cells, noted as $T_{e\_T + }$ and $T_{e\_T - }$.

In this study, we take the empirical data collected from the wet lab as ground truth. For the evaluation of the models, we compare the model results against the empirical data. In order to do so, we firstly applied kernel density estimation with using same parameters and approaches we applied in empirical data. Subsequently, with the borders we generated from the empirical data, we calculated the total density within the defined borders on our model results as well. We run each model 100 times to take into stochasticity effects, and calculated the total density of the aggregated 100 runs results from both T+ and T− cells separately noted as $T_{m\_T + }$ and $T_{m\_T - }$. We calculate SCAPD to express the difference observed between the model results and the experimental data (used as ground truth), as follows:

4 $$\begin{array}{*{20}c} {SCAPD = \left| {T_{e\_T + } - T_{m\_T + } } \right| + \left| {T_{e\_T - } - T_{m\_T - } } \right|} \\ \end{array}$$

In the next step, we got the sum of the absolute difference of T+ and T− cells total density within the area for both disc and ellipse colonies. A small sum of absolute total density difference indicates the model is close to the empirical data (if completely matching the empirical data, then the absolute difference would be exactly zero).

## Results

### Ground truth

Using the borders we defined for SCAPD on Fig. 6, we calculated the total density within the areas for T+ and T− cells in the empirical data on both disc and ellipse micropatterns. The results of total density from the empirical data are shown in Table 6.

**Table 6:** Total density within the area from empirical data.

	T− cells	T+ cells
Disc	0.8233	0.7407
Ellipse	0.8471	0.5064

### Basic models

**Figure 7:**
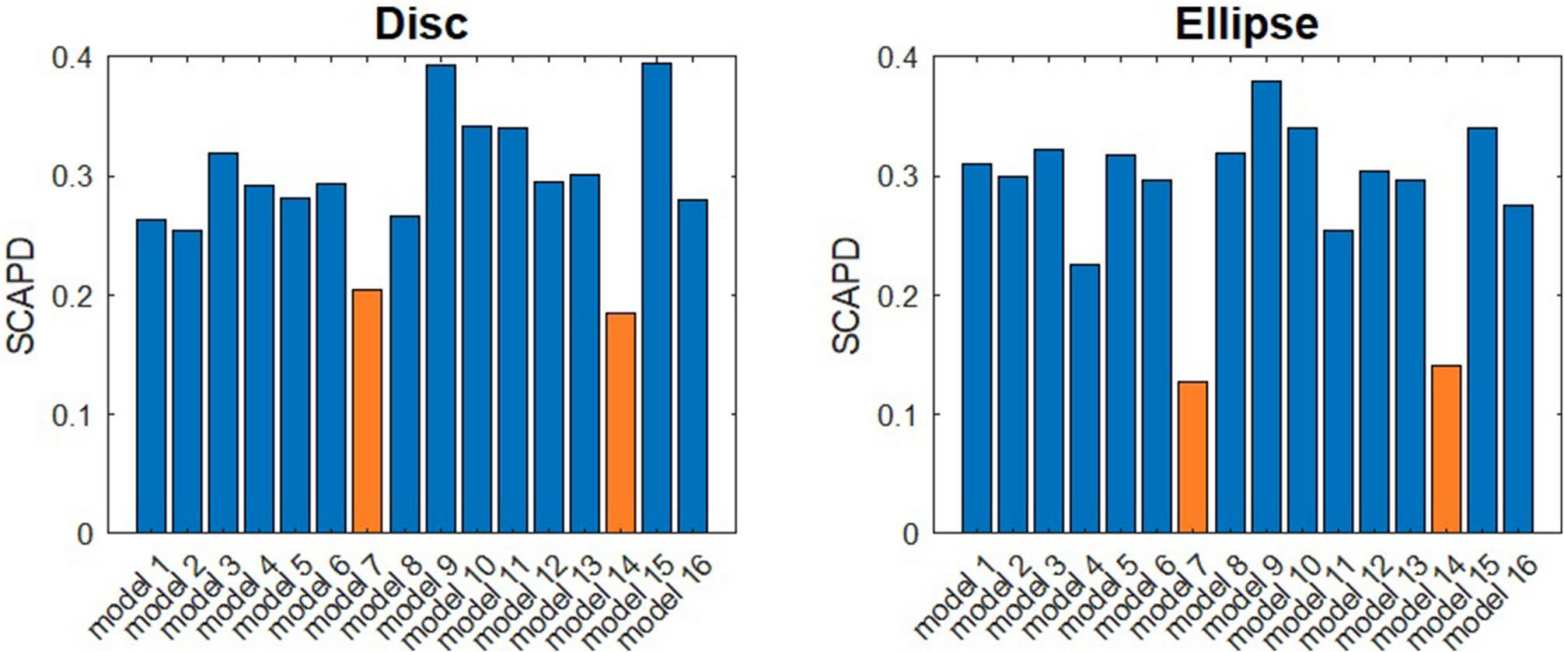
SCAPD results from 16 models for disc and ellipse micropatterns.

On Fig. 7, we show the SCAPD results from 16 models for both disc and ellipse micropatterns. As shown in Fig. 7, for both disc and ellipse micropatterns, Model 7 (with different velocity and directional movements) and Model 14 (with different velocity, directional movements, and border effect) have the best performance. For disc models, Model 14 is slightly better than Model 7, while for ellipse models, Model 7 is slightly better than Model 14. However, the difference between Model 7 and Model 14 is minimal. So in the next step, we focus on Model 7 and Model 14.

We tested the sensitivity of model output with respect to changes of the parameter of angle change in Model 14. As the results shown in Table 7, the model output, based on the results of SCAPD, is not sensitive to the small degree of angle change.

**Table 7:** SCAPD results of Model 14 for disc and ellipse micropatterns with different angle change values, demonstrating the model is very robust to the choice of this parameter.

Angle change value (°)	Disc	Ellipse
10	0.3591	0.6593
20	0.3992	0.6593
30	0.3546	0.6593
40	0.3640	0.6593

### Models with parameter optimisation

**Figure 8:**
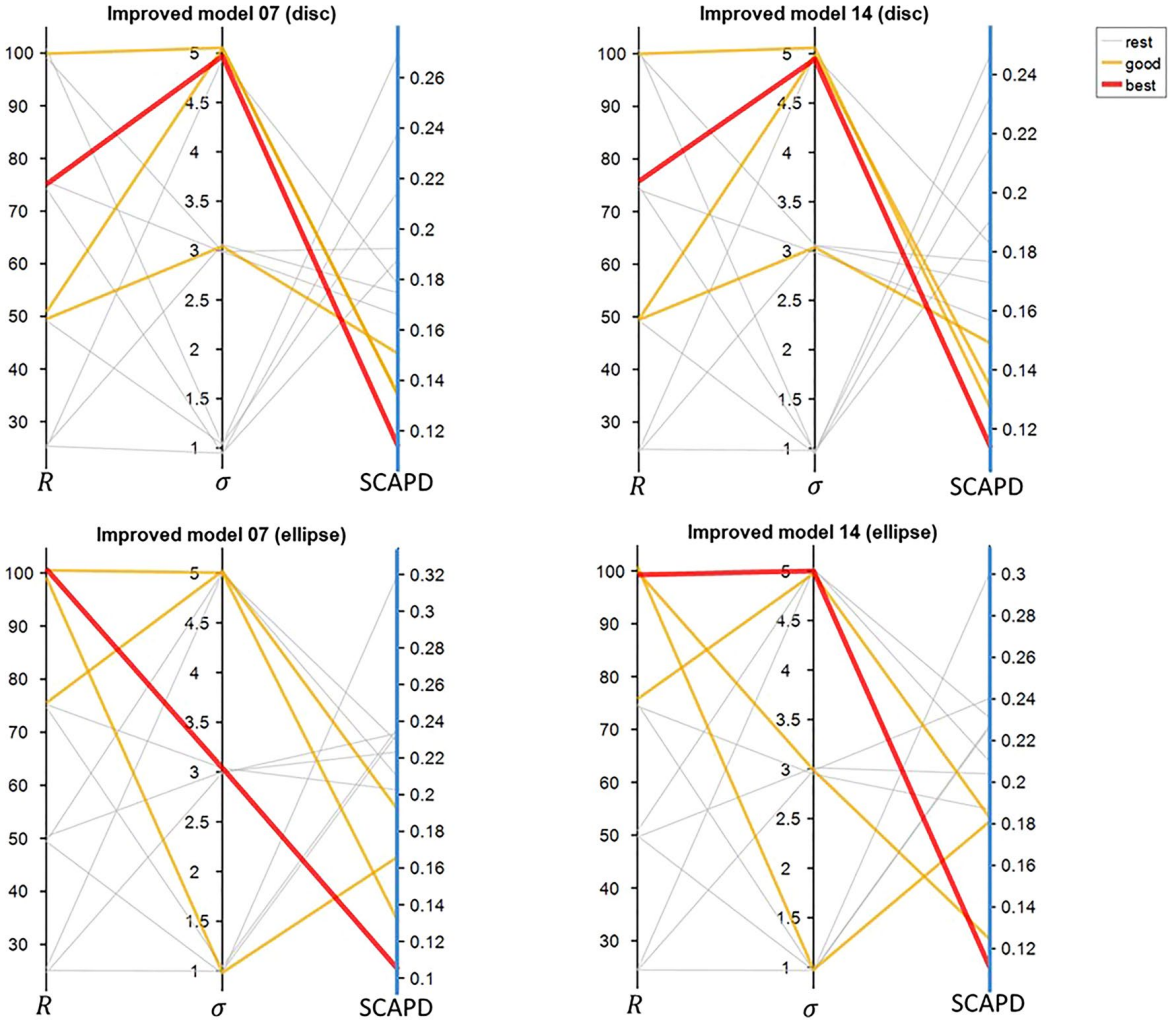
SCAPD results from models with grid search for parameters optimization. Black vertical lines stand for parameters we tested, sensing radius (*R*) and standard deviation (*σ*). The last blue vertical lines show SCAPD results. Each line crossing three vertical lines stand for each mode with specific parameters setting and quantified SCAPD. Red line is the best performance model in this group; orange lines are the following three good models; grey lines stand for the rest models.

Based on the original Model 7 and Model 14, we improved the rules by getting the ratio of velocity. Afterwards, we tested a set of parameters combination of sensing radius (*R*) and standard deviation (*σ*) (the possible values we tested are listed in Table 4) and got the SCAPD results as shown in Fig. 8. For disc models, Model 14 is slightly better than Model 7, while for ellipse models, Model 7 is slightly better than Model 14. However, the difference is extremely small (0.002213 for disc models and 0.0006 for ellipse models). Hence, with getting the ratio of velocity and parameter optimisation through grid search, we reduced SCAPD about 38% from the original best model for disc and about 27% from the original best model for ellipse.

### Best performance models

**Figure 9:**
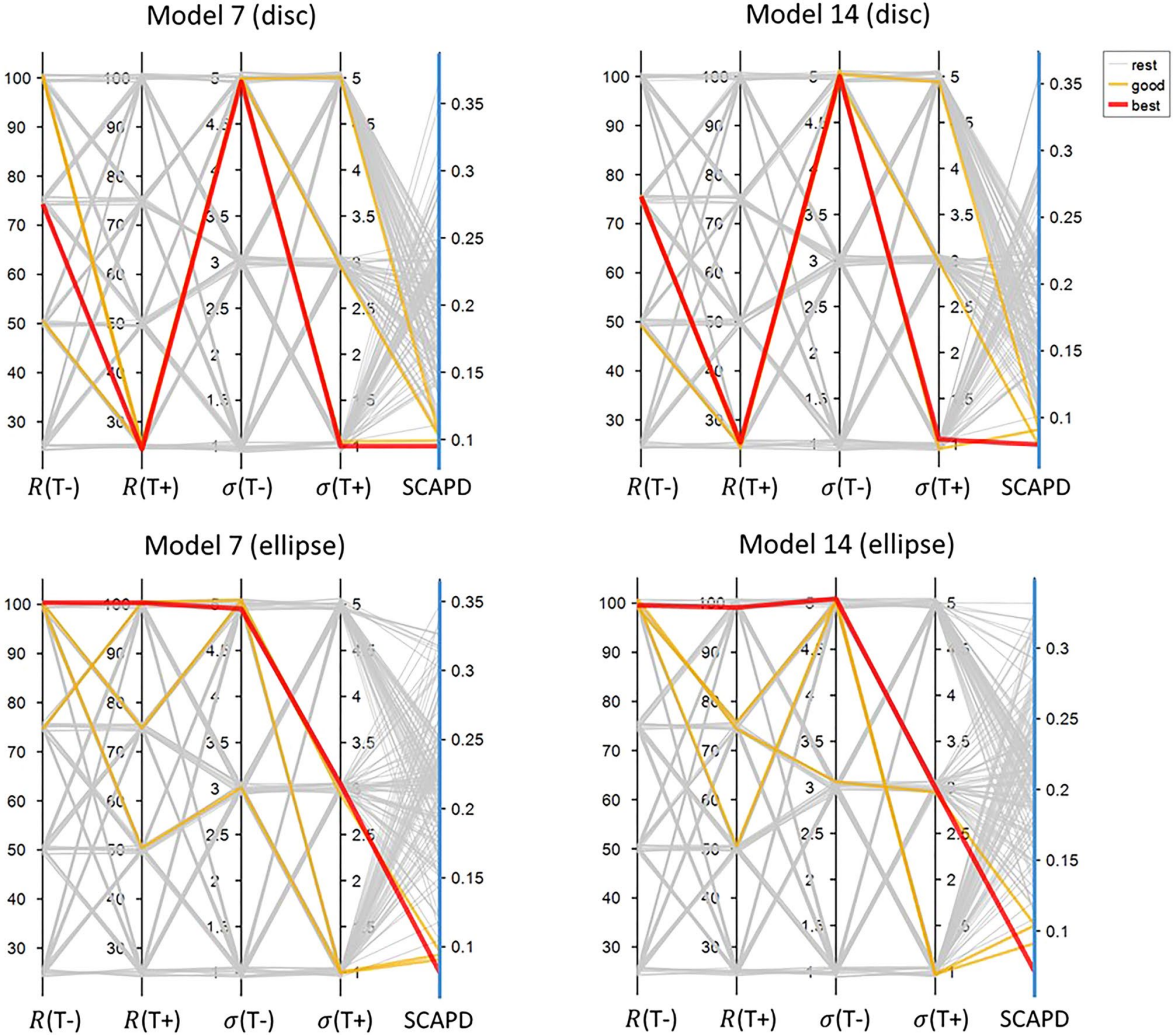
SCAPD results from models with different parameter settings for T+ and T− cells. The structure of the plots is similar to Fig. 8. The first four vertical lines stand for four parameters (different sensing radius (*R*) and different standard deviation (*σ*) for T+ and T− cells).

Subsequently, we tested different sensing radius (*R*) and different standard deviation (*σ*) for T+ and T− cells separately to bring the models closer to the empirical data. Figure 9 shows the results of all combinations of the parameters. As we can see in Fig. 9, sensing radius (*R*) plays an important role to models results, while models are not so sensitive to standard deviation (*σ*). Even though sensing radius should be an intrinsic cell property, we have different optimised values for disc and ellipse micropatterns. This difference might cause by (1) the natural properties of the different shapes of micropatterns since part of the circle that we considered as neighbourhood of the cells would be empty due to part of the circle would be outside of the micropatterns; (2) different crowdedness between different shaped micropatterns. Hence, it is reasonable that disc and ellipse micropatterns have different optimised values for these parameters.

Table 8 summarizes the final SCAPD results from the best performance models compared to the initial random models (Model 1 without any specific motility rules in the original 16 models). Since the mathematical properties of disc and ellipse, we took the results from the random model as the baseline and checked the improvements of our models by comparing against the initial random models. The improvements in our models reach up to 70% and 77% performance improvements in terms of SCAPD for disc and ellipse micropatterns respectively.

**Table 8:** SCAPD results from random models and best performance models.

	Disc	Ellipse
Random model	0.26	0.31
Best performance model	0.08	0.07
Improvement	70%	77%

Finally, we wanted to assess the computational time required to test each model. We tested out the models on a high performance compute cluster with requesting 64 GB memory. We repeated the timing computations 100 times for each model. The total time consumption for each model is listed on Table 9.

**Table 9:** Time consuming of running model 7 and Model 14 for disc and ellipse micropatterns.

Models	Total running time (22,500 runs) (min)	Average running time per run (min)
Model 7 (disc)	800	0.0356
Model 7 (ellipse)	1970	0.0876
Model 14 (disc)	910	0.0404
Model 14 (ellipse)	23,225	0.1033

## Discussion

Previous studies on cell sorting and tissue morphogenesis have described motility driven pattern formation and provided some insights at the molecular level^31^. In this study, we focused on specific pattern formation in ESCs and constructed 16 agent-based stem cell pattern formation models to test all combinations of four biologically plausible rules of cell motility that we have defined. Furthermore, we introduced a new metric, SCAPD, quantifying differences between the model derived pattern formation and the experimental ESCs pattern formation. We found that Model 7 and Model 14 have best performance, which is consistent for both disc and ellipse micropatterns. Model 7 is built using different velocity and directional movements, while Model 14 has an additional rule compared to Model 7 also including the border effect.

We applied grid search for parameter optimisation to bring our model outputs closer to the empirical data. We tested different values of sensing radius and standard deviation. We also tested different values for angle change in rule iv and proved that the model output is not sensitive to this parameter. In consequence, we observed that SCAPD of Model 7 and Model 14 after parameter optimisation is considerably lower compared to the original outputs. Specifically, we computed SCAPD as 0.08 and 0.07 for disc and ellipse micropatterns respectively after revising rules and parameters optimization. Compared to the initial random models, the models’ performance was improved by 70% and 77% for disc and ellipse micropatterns, respectively. The presented models can probabilistically produce broadly realistic pattern formation (when compared to the empirical data).

Different modelling methods have been applied to study stem cells at a population level to reproduce the population dynamics by generating minimal models^32^. For example, Libby and colleagues have applied cellular Pott models to human pluripotent stem cells, enabling a machine learning optimisation approach to predict experimental conditions that yield targeted multicellular patterns^33^. Multiple mathematical models were applied to increase the precision of modelling stem cell proliferation^34^ and investigating stem cells self-renewal^35^. Compared to cellular Pott models, which is targeted to achieve the pattern emergence with the possible simplest computation, agent-based modelling provides higher freedom as cells are free to move and cells can interact with other cells and their environment. Hence, agent-based modelling is a suitable modelling method for investigating cell motility. In addition, agent-based modelling have been widely applied in cell biology. For example, Briers and colleagues applied agent-based modelling to study specific pattern formation in ESCs differentiaion^36^. In this study, we applied agent-based modelling to investigate specific observed pattern formation in ESCs with focusing cell motility, and tested a wide range of motility rules to obtain the minimal rules that can reproduce the pattern formation. Agent-based modelling allows us to model cell motility intuitively and gives insight of cell motility.

Compared to our previous work, in this paper, we tested all combinations of the proposed biological plausible rules and improved the models performance to a better level based on the results from the new proposed metric SCAPD. As one of the challenges of applying agent-based modelling in morphogenesis is the evaluation of patterning^37^, in this study, we tested multiple existing metrics for evaluation, including earth mover’s distance, Kullback–Leibler divergence, and continuous rank probability score, and demonstrated that a new metric is required. SCAPD evaluates the models’ results by calculating the distance between models’ results and empirical data. Based on SCAPD, we quantified our models’ results with different rules instead of visually estimating the results. The high variety in empirical data could be the reason that existing approaches for evaluation resulting outcomes that do not follow the visual impression we had from the resulting patterns.

The cell behaviours in reality may differ in practice from the simplifying assumptions explored in the context of this study. However, we demonstrated that we could replicate the pattern formation with a quantified level of uncertainty. Firstly, we proposed new testable rules for understanding the mechanisms of pattern formation based on the presented work. The new hypotheses are that the pattern formation can be achieved by engineering cell speed and the level of adherence of cells by using synthetic biology^38,39^. These hypothesis could be verified through experiments in the future. Secondly, these findings can support the future study on engineering cell motility to obtain desired patterns. Lastly, we can propose potential initial states for desired patterns by finding the probabilistic corresponding relationship between initial states and the final states in our models. More experiments would be required to verify the corresponding relationship. Through these experiments, we provide contributions to regenerative medicine by increasing the robustness of achieving desired pattern formation and having a better understanding of the driving power of pattern formation from population level.

In this study, we generated probabilistic models to reproduce the observed pattern formation in pluripotent stem cells. We developed a new metric SCAPD to evaluate the models’ results at a quantified level. Through revising cell motility rules and parameters optimization, we improved the models performance about 70% and 77% compared to initial random model for disc and ellipse micropatterns respectively. The models provide opportunities for engineering the cells in reality to achieve the desired pattern formation. Overall, we see this study as a step towards extending the current understanding of ESCs pattern formation, which may facilitate the development of novel stem cell therapies.
